# What Does Work Signify for Those in Search of Labor? Meaning of Work for the Unemployed Who Attend an Employee Orientation Program

**DOI:** 10.3389/fpsyg.2018.01788

**Published:** 2018-09-26

**Authors:** Yolanda Navarro-Abal, José Antonio Climent-Rodríguez, María José López-López, Juan Gómez-Salgado

**Affiliations:** ^1^Department of Social, Developmental and Education Psychology, Faculty of Education Science, University of Huelva, Huelva, Spain; ^2^Department of Clinical and Experimental Psychology, Faculty of Education Science, University of Huelva, Huelva, Spain; ^3^Department of Nursing, University of Huelva, Huelva, Spain; ^4^Universidad de Especialidades Espíritu Santo, Guayaquil, Ecuador

**Keywords:** unemployment, meaning of work, work centrality, employee orientation programs, employment, factor analysis

## Abstract

**Introduction:** Work is one of the most important areas in people’s lives. This is highly related to the meaning of work people possess and the social culture that surrounds them. However, unemployment stands out as a major social phenomenon of the 21st century of concern for governments, institutions, and professionals, generating the need to reflect, among other issues, on the processes that favor and keep the person in the situation of unemployment, and to think about the real effects of the measures aimed at supporting and guiding the unemployed. The objective of this work is to analyze the existing differences in relation to societal norms, values, and work centrality in unemployed people who attended employment guidance with respect to another group of unemployed people who didn’t received this guidance.

**Method:** A descriptive, analytical, cross-sectional study was conducted. The sample consisted on a total of 227 users of the Employment Guidance Service Center of the province of Huelva, who were divided into two groups. A first group of 112 users who make use of the employment guidance programs of the Andalusian Public Employment Services, and a second group, or control group, made up of 115 unemployed people who do not make use of these services. The MOW International Research Team questionnaire has been used for the assessment of the participants.

**Results:** In relation to societal norms, people who do not place their trust in employment guidance conceptualize work as a right and not as a duty; in terms of work values, those with lower levels of training value professional status and prestige with higher scores, whereas people with a higher level of training value the satisfactory nature of work. Regarding centrality, high-level results are obtained throughout the sample, and it ranks second only to family when assessing relative centrality.

**Conclusion:** It can be seen how some variables will determine a greater or lesser level of motivation toward the search for employment. This work should lead to a reflection on the need to generate employment insertion programs that are more adapted to the idiosyncratic characteristics of individuals and groups.

## Introduction

Unemployment is one of the social phenomena causing greater concern to citizenship and Spanish public managers today. The economic crisis scenario has brought unemployment rates unknown to date, that widely exceed 20% of the working population and reach more than 50% among the younger population in search of employment. In parallel, this serious situation has generated the need to reflect on the suitability of different policies, methodologies, techniques, and tools related to job search and the inclusion of unemployed people. Among these, employment guidance holds a special place, being an element of support and intervention for unemployed people that allow them to work on technical, emotional, competence, and strategic aspects related to the improvement of their employability (Jiménez, 2017).

Some authors such as Pérez de las Vacas (2015) emphasize the importance of the unemployed in the job search process, making special mention of the person-focused psychosocial variables. These include motivation and attitude during the job search, expectations of control perceived about it, locus of control, both internal and external, and expectations of success. Many of these variables are directly related to people’s work values and the meaning they attach to working. Based on these personal “good practices” to find employment, the author describes the elements that should be “mobilized” by employment consultants in the counseling process; among them, to define work goals, identify the work they want to perform, and plan the search for and access to employment. To do this, it is necessary to get to know their personal qualities and professional preferences, and to rely on the fact that the person identifies the requirements for the performance of the profession and is aware of the surrounding socio-economic environment. However, it is a reality that employment guidance does not, by itself, increase the employability and chances of finding a job in all cases. The possible positive effects of this counseling depend on numerous variables regarding the technical characteristics of the intervention and the professional profile, motivation, and certain psychosocial variables of the unemployed person (Piqueras, 2013). In particular, proponents that highlight certain psychosocial variables as especially relevant for finding a job are numerous (Montilla, 2003; De Pablo-Urban, 1996). Among these, centrality appears recursively, understanding that those people who have a higher level of work centrality, among others, will have a better prognosis in their search for employment, meaning that they will find a job before those who do not possess this high centrality. This is, therefore, related to the person’s active search for employment, as well as the fact that confidence and motivation during this search will provide positive results.

Work is, at present, one of the most important aspects in people’s lives and it fulfills a number of functions, both positive and negative, for the individual. Thus, some authors have concluded that it is a source of satisfaction (Dakduk and Torres, 2013), passion, and involvement (Zigarmi et al., 2009), as well as discomfort (Gil-Monte, 2003). Meanwhile, Ruiz-Quintanilla and Claes (2000) indicate that work provides status and social prestige, professional and personal identity, interesting social contacts, satisfaction, and economic autonomy, at the same time that it is related to other basic functions for the individual and society. Work constitutes a means of economic survival and a developmental factor of the social, political, cultural, and personal life that acts, also, as a source of health and illness, wellness, and physical, psychic, and social unrest (Blanch, 2011). These functions of work are highly related to the meaning of work people possess and the social culture that surrounds them.

The *meaning of work* consists of a set of beliefs, values, and attitudes toward working. This meaning is learned before and during the process of socialization at work, and varies depending on the subjective experiences and situational issues that occur at work and within its organizational context (Salanova, 1992). It is a multidimensional construct, something that makes its conceptualization and operationalization difficult. The Meaning of Working [MOW] and International Research Team, 1987 distinguishes three main dimensions: work centrality, societal norms, and work values. The dimensions of the meaning of work have different degrees of stability; and this approach focuses on the entire meaning of work.

A somewhat different perspective consists of attending to its different components such as dimensions with degrees of stability that are not necessarily identical. Thus, Selva (1988) considered work centrality and societal norms as the basic components of the socialization process, i.e., they would form before the subject’s commencement to work, during the stage that is commonly called “anticipatory socialization” or “work socialization.”

Regarding centrality, as designated by Vanderberg and Self (1993), some studies claim that it decreases during the first 6 months of employment, while others point out that six 6 later, i.e., a year after being in the organization, an increase of the same occurs.

*Work centrality* has been defined as the degree of general importance that working has in one’s life at any given time, i.e., to the extent that this is central to their personal identity (Sverko, 1989). This construct has been widely investigated by the Meaning of Working [MOW] and International Research Team, 1987, who defines it as a general belief about the value of work in a person’s life and as the degree of general importance working has at any given time. These authors operationalize this construct in two ways, distinguishing absolute centrality from relative centrality. The first tries to determine the overall importance of work in the individual’s life; and the second determines the importance attached to work in relation to that given to other relevant areas of life such as family, leisure, religion, or participation in the community’s social life. Authors such as Gracia et al. (2001) discuss the meaning of conceptual differences between involvement, engagement, and work centrality, and the way in which they have been used in empirical research under the basis of the existence of a common construct. Salanova et al. (1996) distinguish centrality from job involvement and commitment to work. They claim that the first basically refers to a belief that individuals have regarding the role that work has in their lives, while the involvement and the commitment to work put an emphasis on cognitive, affective, and emotional responses, as well as on the attitudinal and behavioral implications they have for people’s performance. On the other hand, Kanungo (1991) indicates that two components are distinguished within work centrality: a normative belief about the value of work in one’s life (work-role centrality), and the centrality of work as an activity now regarding the other spheres of life.

On the other hand, *societal norms* applied to work are described as claims that people use when they perform regulatory assessments of work (Meaning of Working [MOW] and International Research Team, 1987), or also claims relative to fairness at work, from the individual’s perspective and from society’s perspective. More specifically, it refers to the extent to which subjects are in accordance with a series of statements about the rights and duties toward working.

The third component of the concept of the meaning of the work is *work values*. The values which people acquire and develop at work represent an important component of the meaning of it, together with the regulatory aspects and beliefs about it and the centrality it occupies in their lives. Schwartz (1992) refers to values as the desirable state, objectives, goals, or behaviors that go beyond specific situations and that are applied as standards for judging and choosing between alternative modes of behavior. Attitudes, on the other hand, are beliefs about specific objects or situations, and they can be positive or negative (values are only positive). Due to its specificity, the interests are placed between the values and the attitudes.

*Work values* refer to what people expect to obtain in exchange for their dedication to work. Hoppock and Super (1950) gave us a first approach to the concept of work values when they observed that generalized expressions on work satisfaction tended to relate to expressions of satisfaction toward specific aspects of work such as salary, schedules, promotion, diversity, etc. Later on, Super (1968) considered work values as the goals that motivate individuals to work: those goals could be intrinsic or extrinsic to work. For Zytowski (1970), work values are regarded as concepts that mediate between affective counseling for people and the different kinds of external objects offering similar satisfactions. In addition, Pryor (1981) considered that work values represent qualities or specific rewards that one wants to obtain from work (for example: money, altruism, etc.).

The Meaning of Working [MOW] and International Research Team has addressed the analysis of work values by distinguishing two constructs: the valued work outcomes and the importance of the different work aspects for people (García-Montalvo et al., 2003). The work values or the valued work outcomes have traditionally been estimated as motivators of the human behavior (Salanova et al., 1993). The valued work outcomes act as a guide and are identified as the reasons for working; they are the set of results that the person seeks in “working” and constitute the basic reasons why people work. The second construct, which we can call “importance of work goals,” consists of the importance that people give to different work aspects and should be understood as the updating or concretion of values in a given work (Salanova, 1992), i.e., what subjects prefer to find in a particular job.

Considering the characteristics previously analyzed in relation to the concepts of centrality and meaning of the work, all the dimensions are formed by different subscales that group a certain number of items which measure the same variable. Only in the case of labor goals, the items are not grouped in subscales or subdimensions; therefore, the first objective of this work is to carry out an exploratory factor analysis to find possible homogeneous groups of these variables.

In addition, other objectives of this research are to describe and compare the social norms, values, and relative and absolute work centrality in a sample of unemployed people who attend an employment guidance service, and another group of unemployed people who do not make use of this service.

## Materials and Methods

### Participants

The sample consists of a total of 227 participants, of which 73 are men (32%) and 154 are women (68%). The age range is between 18 and 52 years (*M* = 28.51, *TD* = 9.70).

The sample is divided into two groups of unemployed people. The first of these was formed by 112 people attending employment guidance programs of the Andalusian Public Employment Service. The participants have an age range between 22 and 52 years (*M* = 36.31, *TD* = 7.13), with 56.30% of women and 43.70% of men.

The second group is the control group, consisting of a total of 115 participants in an unemployment situation who do not attend the employment guidance services. The age range is between 18 and 46 years (*M* = 20.98, *TD* = 4.5), mostly women (79.3%), compared to the number of men (20.7%).

### Instruments

The following instruments have been used for the assessment of the participants:

- Socio-demographic variables through a protocol for data collection prepared *ad hoc* by the research group that developed this work (age, sex, time in unemployment, level of studies, and income of the family unit).
- Meaning of Working [MOW] and International Research Team (1981, 1987) adapted by Salanova et al. (1987), whose main objective is to assess the meaning of working. To analyze the empirical structure of this tool, two investigations with different samples were carried out. A first investigation with a sample of 8700 participants from eight different countries, and a second, with a sample of 5800 workers representing ten different categories. For the analysis of the instrument, a study of the main components was carried out on 37 of the 39 central items. Initially, that instrument was made up of five different dimensions: *centrality, societal norms about working, valued work outcomes, work goals, and identification with the job role*. Regarding the last dimension, given the little information the questionnaire provided on it, it was subsequently eliminated from the instrument. These dimensions do not operate in a person independently, but they configure different gestalts or patterns. However, it is possible to assess each of them by allowing independent assessments.

In this way, in this work, we have assessed the other four dimensions: centrality, societal norms about working, valued work outcomes, and work goals. Since the authors themselves show the little relevance of the identification dimension with the work role, and considering that the sample was made up of unemployed people, the assessment of that dimension has not been carried out, that is, the measurement of the degree in which one identifies with the working activity, either with the tasks carried out, the organization to which they belong, etc. The dimensions of the instrument used are the following:

(1) Work centrality: composed by two subdimensions: centrality in absolute terms or absolute centrality, and centrality in relative terms or relative centrality. To measure the former, the questionnaire has a component of belief/value, understanding centrality as a life role, i.e., the assessment between personal identity and professional identity. It is measured through the question: How central is work in your life? With a Likert-type response of 1 (work is one of the least important things in my life) to 7 (work is one of the most important things in my life). The second subdimension, relative centrality, assesses the counseling/decision component. To do this, the person will have to compare the working sphere with the four spheres of life (leisure, religion, family, and community) proposed by Dubin (1956, 1976). The participant must distribute 100 points among the five spheres.
(2) Societal norms about work: it assesses the normative reasoning that acts as the antecedent of the social principles and behaviors. It is formed by two subscales: employment guidance as a right and employment guidance as an obligation, with a Likert-type scale of 5 points.
(3) Valued work outcomes: dimension that refers to the functions perceived during the working activity and that is valuable for the individual. The participants indicate which outcomes they seek to obtain from working, as well as their relative importance, distributing a total of 100 points among the six values associated to work (status and prestige, needed income, time absorption, interesting contacts established and maintained at work, opportunity to serve society, and professional interests and satisfaction). It distinguishes between an instrumental or extrinsic assessment of the work outcomes and an expressive or intrinsic assessment of the same. The first expresses the instrumentality of work, that is, the means to achieve an end. The second indicates an appraisal of work as satisfactory in itself that contributes to the person’s feelings of personal and work self-fulfillment, competence assessment, and self-determination. Also, it is worth valuing work as the opportunity to establish interesting social contacts or satisfactory interpersonal relations, a consequence of the connection with other people through work relations (companions, managers, clients, etc.).
(4) Work goals: it refers to the relative importance of the objectives that individuals prioritize when they perform a particular work, and would explain the main motivations for working. In this dimension, aspects related to the reasons why people work are not assessed, but what people expect to find or get from work. As the authors of the MOW group point out, these two dimensions are not in contradiction, but complementary. Why a person works and what he/she wants to get from work are related issues, though not identical. In this sense, the instrument assesses 15 goals by means of a Likert-type scale from 1 (not important) to 5 (very important). The variables included are: opportunities for learning, interpersonal relationships, opportunities for advancement or promotion, adequate working hours, variety, interesting work, job security, match between job requirements and one’s abilities and expertise, salary, conditions, autonomy, new challenges, power, possibilities for innovation, and occupational fulfillment.

### Procedure

In the group of unemployed people who attend the employment guidance services, the data collection was carried out by offering all the unemployed that accessed the employment guidance service of the Andalusian Public Employment Service to take part in the study. When they attended the scheduled appointment for their orientation process, they were explained the purpose of the research and offered the possibility of participating in it. In case of acceptance, they were administered the questionnaires individually in an independent room, to ensure the correct understanding of the instructions. The confidentiality of the data was assured, and participation was voluntary.

The group of unemployed people who do not attend the employment guidance services was selected by offering the participation in the study to the users of the offices of the Andalusian Public Employment Service. As in the previous case, when they attended the scheduled appointment, questionnaires were administered individually, and the participation was voluntary and confidential.

#### Ethical Approval Procedures

The study was conducted in accordance with the Declaration of Helsinki; the procedure for carrying out this research work was analyzed and ratified by the Provincial Commission of the Andalusian Employment Service (SAE).

This procedure was carried out through the Provincial Commission of the SAE in whose facilities this research has been carried out. This Commission is a body dependent on the regional government of Andalusia and maintains professional relations with the University. It operates as an Institutional Review Board that ensures the good functioning of the public institution and performs, among other functions, the planning, management, promotion, and evaluation of the different programs and actions for employment in Andalusia. In particular, it is responsible for monitoring and evaluating the activities of the SAE Agency and proposing the measures it deems appropriate to ensure good praxis, ethical, and deontological adequacy.

Since this Commission was in charge of reviewing and approving this research, the University of Huelva, the institution of the authors, did not act as an evaluation committee nor did it require its own approval for the development of this project. The investigators signed their respective documentation facing the members of the Provincial Commission of the SAE to ensure their commitment to the anonymity of the sample and respect for their rights.

Likewise, the inclusion in the study was carried out ensuring the willingness to participate, the complete information about the process and the secrecy of confidentiality of the interviewers. For this, the consent obtained from the interviewees was both informed and written to ensure the correct ethical procedure.

### Data Analysis

In order to verify the factorial structure of the instrument used, an exploratory factor analysis was conducted. For the extraction of data, the method of *main components* and Varimax rotation were used. This method identifies factors with high or non-existing saturations within the variables. Therefore, there were factors with high correlations and a small number of variables, and zero correlations in the rest; thus, the factor variance is redistributed. The sample adequacy was measured by the sample adjustment tests [Kaiser–Meyer–Olkin (KMO) index and the Bartlett sphericity test].

For the comparison of the metric variables, after verifying the normality by means of the Kolmogorov test, means contrast analysis, and specifically student’s *t*-test were performed. Information on effect size tests is provided using the Cohen’s *D*-test, interpreted according to the following criteria: from 0 to 0.19, very small effect size; from 0.20 to 0.49, small effect size; from 0.50 to 0.79, medium effect size; and from 0.80, large effect size (Cohen, 1988; Huberty, 2002).

## Results

An analysis of the internal structure presented by the MOW International Research Group instrument was carried out, first, in order to find out whether participants’ responses were grouped into variables that had a common meaning and that could reduce the number of dimensions needed to get as much information as possible from the answers. More specifically, a factorial analysis of the *work goals* scale scores was carried out, being this the only one that does not group the items in categories or subscales. Suitable indexes (KMO = 0.873) and Bartlett’s test of sphericity [*x*^2^_(105)_ = 995,601, sig < 0.01] are available for the adjustment of the sample suitability.

According to the estimation of the factors criterion (values higher than 1), in this work, the results show the grouping of the items into four factors, which explains 57.44% of the total variance. In **Table 1**, the values of the “self-values” and the explained variance of each factor are found. Factorial scores in each of the factors show no significant difference between the two groups.

**Table 1:** Retained factors and total variance explained.

Components	Initial eigenvalues			Extraction sums of squared loadings		
	Total	% of variance	Cumulative %	Total	% of variance	Cumulative %
1	5.30	35.35	35.35	5.30	35.35	35.35
2	1.19	7.98	43.33	1.19	7.98	43.33
3	1.09	7.31	50.65	1.09	7.31	50.65
4	1.01	6.79	57.44	1.01	6.79	57.44
5	0.97	6.50	63.95			
6	0.74	4.96	68.91			
7	0.71	4.78	73.70			
8	0.64	4.31	78.01			
9	0.61	4.09	82.11			
10	0.56	3.76	85.88			
11	0.53	3.59	89.47			
12	0.46	3.09	92.56			
13	0.42	2.80	95.37			
14	0.38	2.56	97.94			
15	0.30	2.06	100.00			

To analyze the rotated components matrix, factorial loads lower than 30 were eliminated. In this simplified matrix, we can observe the relation between each factor’s items: factor 1, “work as a challenge,” explains 35.35% of the variance; factor 2, “working conditions,” explains 7.98% of the variance. On the other hand, factor 3, “development of personal relationships,” explains 7.31% of the variance and, finally, factor 4, “status and prestige,” explains 6.79% of the variance.

As shown in **Table 2**, the grouping of the items in the found factors is distributed as follows: In factor 1, items grouped are *autonomy, variety, performing an interesting work, possibilities for constantly setting new challenges and improve oneself to achieve them, and possibilities for innovation*.

**Table 2:** Rotated components simplified matrix.

	Components			
	1	2	3	4
AUTONOMY (decide how work is done)	0.71			
High VARIETY	0.67			
Perform an INTERESTING work (like the job)	0.62			
Possibilities for constantly setting NEW CHALLENGES and improve oneself to overcome them	0.56			
Possibilities for INNOVATION (doing new things)	0.62			
Adequate WORKING HOURS		0.53		
Good opportunities for ADVANCEMENT and PROMOTION		0.44		
Good job SECURITY		0.76		
Good MATCH between job requirements and one’s abilities and expertise		0.54		
Good SALARY		0.61		
Good physical working CONDITIONS (temperature, cleanliness, low noise levels, etc.)		0.68		
Opportunities for LEARNING new things			0.82	
Interesting INTERPERSONAL relationships (supervisors, workmates, etc.)			0.76	
POWER (influencing others)				0.75
Occupational FULFILLMENT (achieving social prestige)				0.74

Factor 2 includes the items: *adequate working hours, good promotion and advancement opportunities, job security, good match between job requirements and one’s abilities and expertise, training and experience, good salary, and good physical working conditions*.

Finally, factors 3 and 4 group the following items: *opportunities for learning new things and interesting interpersonal relationships* in factor 3, and *power and recognition* in factor 4.

The following are the differences for each dimension of the MOW International Research Group between the two groups studied (**Table 3**). In the *centrality* dimension, there are statistically significant differences in the items *absolute centrality* [*t*_(226)_ = 4.36, *p* < 0.01, *d* = 0.58], where the group of participants who attend the employment guidance services present the higher scores.

**Table 3:** Descriptive statistics and means comparison between groups regarding the dimensions centrality at work, societal norms about working, and valued work outcomes.

	Group	*M*(*SD*)	*t*	*d*
**Centrality**
Absolute	UAJC	6.03 (0.97)		
	U	5.41 (1.12)	4.36*	0.58
**Relative**
My leisure time	UAJC	16.83 (9.90)	3.66*	0.48
	U	23.34 (16.00)		
My community participation	UAJC	7.53 (8.77)	2.07*	0.27
	U	10.76 (12.70)		
My work	UAJC	29.61 (15.11)	3.33*	0.44
	U	22.99 (13.85)		
My religion	UAJC	3.66 (6.85)	0.79	
	U	4.66 (10.26)		
My family	UAJC	42.42 (15.33)	2.97*	0.39
	U	35.42 (18.02)		
**Societal norms**
Work as a right	UAJC	19.87 (3.62)	0.45	
	U	20.08 (3.43)		
Work as an obligation	UAJC	18.25 (3.54)	0.40	
	U	18.42 (2.94)		
**Valued work outcomes**
Status and prestige	UAJC	10.57 (10.30)	2.55*	0.34
	U	14.77 (13.68)		
Needed income	UAJC	38.35 (19.81)	5.61*	0.74
	U	24.76 (15.00)		
Time absorption	UAJC	13.57 (8.61)	0.93	
	U	12.15 (13.36)		
Allows maintaining interesting relationships with others	UAJC	12.52 (9.73)	1.89	
	U	15.76 (14.96)		
Opportunity to serve society	UAJC	10.13 (7.15)	3.17*	0.42
	U	14.93 (14.17)		
Working is interesting and	UAJC	15.71 (10.68)	1.58	
satisfactory in itself	U	18.47 (14.63)		

*UAJC, unemployed attending job counseling (*N* = 112); U, unemployed not attending job counseling (*N* = 116). ^∗^*p* < 0.01*.

Regarding *relative centrality*, statistically significant differences are found in all items except for religion. The group of unemployed people who attend the employment guidance services present higher scores in the items *work* [*t*_(226)_ = 3.33, *p* < 0.01, *d* = 0.44] and *family* [*t*_(226)_ = 2.97, *p* < 0.01, *d* = 0.39]. In the items *leisure* [*t*_(226)_ = 3.66, *p* < 0.01, *d* = 0.48] and *community participation* [*t*_(226)_ = 2.07, *p* = 0.03, *d* = 0.27], the group of participants who do not attend employment guidance obtains the higher scores.

As for the dimension *societal norms about work*ing, there are no statistically significant differences in any of the two subscales. Therefore, there is no difference between both groups in the dimensions *employment guidance toward work as a right* [*t*_(226)_ = 0.45, *p* = 0.650] and, the other, *toward work as an obligation* [*t*_(226)_ = 0.40, *p* = 0.684].

In relation to the data obtained in the dimension *valued work outcomes*, there are statistically significant differences in the items *status and prestige* [*t*_(226)_ = 2.55, *p* = 0.011, *d* = 0.34], *needed income* [*t*_(226)_ = 5.61, *p* = < 0.01, *d* = 0.74], and *opportunity to serve society* [*t*_(226)_ = 3.17, *p* = < 0.01, *d* = 0.42], obtaining the unemployed group that do not attend employment guidance the highest score on all items except in the item *needed income*.

The comparisons between the two groups in relation to the *work goals* dimension are shown in **Table 4**. There are statistically significant differences in the items *promotion*, *conditions*, and *occupation fulfillment*. The unemployed group that does not attend the employment guidance services presents higher average scores in the items *good opportunities for advancement and promotion* [*t*_(226)_ = 2.53, *p* = 0.01, *d* = 0.34], *good physical working conditions* [*t*_(226)_ = 3.31, *p* < 0.01, *d* = 0.44], and *occupational fulfillment* [*t*_(226)_ = 2.26, *p* = 0.02, *d* = 0.30].

**Table 4:** Descriptive statistics and means comparison between groups regarding the dimension work goals.

	Group	*M*(*SD*)	*t*	*d*
Opportunities for LEARNING new things	UAJC	4.29 (0.77)	0.56	
	U	4.23 (0.76)		
Interesting INTERPERSONAL relationships	UAJC	4.09 (0.81)	1.58	
	U	4.25 (0.75)		
Good opportunities for ADVANCEMENT and PROMOTION	UAJC	3.88 (0.85)	2.53*	0.34
	U	4.16 (0.82)		
Adequate WORKING HOURS	UAJC	3.96 (1.00)	0.14	
	U	3.97 (0.87)		
High VARIETY	UAJC	3.62 (0.96)	0.54	
	U	3.68 (0.90)		
Perform an INTERESTNG work	UAJC	4.37 (0.83)	1.51	
	U	4.53 (0.75		
Good job SECURITY	UAJC	4.54 (0.70)	0.67	
	U	4.48 (0.68)		
Good MATCH between job requirements and one’s abilities and expertise	UAJC	4.01 (0.81)	1.50	
	U	4.17 (0.76)		
Good SALARY	UAJC	4.11 (0.84)	0.01	
	U	4.11 (0.79)		
Good physical working CONDITIONS	UAJC	4.06 (0.86)	3.31*	0.44
	U	4.40 (0.66)		
AUTONOMY (decide how work is done)	UAJC	3.60 (0.96)	1.50	
	U	3.79 (0.94)		
Possibilities for constantly setting NEW CHALLENGES and improve oneself to overcome them	UAJC	3.83 (0.99)	1.47	
	U	4.01 (0.81)		
POWER (manage and influence others)	UAJC	2.78 (1.16)	0.39	
	U	2.83 (0.97)		
Possibilities for INNOVATION (doing new things)	UAJC	3.79 (0.94)	1.13	
	U	3.92 (0.85)		
Occupational FULFILLMENT (achieving social prestige and others’ admiration)	UAJC	3.27 (1.09)	2.26*	0.30
	U	3.59 (1.02)		

*Unemployed attending job counseling (UAJC): *N* = 112; unemployed not attending job counseling (U): *N* = 116. *^∗^ p* < 0.01*.

## Discussion

First, an objective of this work regarding exploratory factor analysis is to be able to obtain as much information as possible from the labor goals through a smaller number of variables that interrelate with each other. Obtaining a smaller number of variables represented by its main components provides the advantage of offering very valuable information of the labor goals, distributed in homogeneous variables.

Regarding the rotated components matrix and the factors found, we can observe that the items saturate in four factors. In the first factor, the items are grouped in *autonomy*, *variety*, *performing an interesting role of work*, *possibilities for constantly setting new challenges and improving one to overcome them*, and *possibilities for innovation*. This first factor has been called “*work as a challenge*” and it explains 35.35% of the variance. As we can see, those variables that McClelland (1976) already mentioned in the theory of needs are grouped. The author describes the feelings and needs that lead people to be professionally motivated and successful. More specifically, the variables related to the need for achievement are grouped, where the person develops the desire of excellence in work and assumes it as a responsibility and a goal.

The percentage of variance explained by the second factor is 7.98% and it has been called “*working conditions*.” It groups *adequate working hours*, *good opportunities for advancement and promotion*, *job security*, g*ood match between job requirements and one’s abilities and expertise*, *good salary*, and *good working physical conditions*. As can be seen, all variables are related to working conditions as well as to other individual variables such as adjustment and imbalanced expectations; more specifically, organizational aspects that facilitate people’s effort and performance, as well as other more intrinsic incentives (Reiss, 2012; Panagopoulos, 2013).

The third factor has been called “*development of personal relationships*,” explaining 7.31% of the variance. This category includes the opportunity for learning new things as well as interpersonal relationships. Again, mention should be made of the McClelland Theory of Needs. In this case, the variables are grouped around the need for filiation, i.e., the need for people to relate to others and seek friends.

Finally, the fourth factor, called “*status and prestige*,” includes the variables power and occupational fulfillment, and explains 6.79% of the variance. Here, the variables are grouped around the power of and the need for occupational fulfillment, understood by McClelland (1961) as the need to get other people to behave in a way that they would not, i.e., it refers to the desire to have an impact on people, to influence and control others. It also refers to the status and prestige it means for people to have a job that dignifies them.

On the other hand, for decades, the suitability of talking about the meaning or the centrality of work has been questioned, based on the great political, economic, and social transformations which even predict the “end of the working society” (Offe, 1992; Gorz, 1997; Méda, 1998). This perspective argues that work has ceased to play a decisive role in the shaping of individual and collective identities. However, the results of our study coincide with other similar ones made in recent years (Gallardo, 2011) in which we observe the high value that the studied subjects attributed to work in their lives; and without distinction of sex, age, level of studies, marital status, or social class. In any case, in our study, and even with all subjects allocating high values to work, it is observed that this assessment is greater among those unemployed people who attend employment guidance services, to the detriment of those others that, although in search of employment, are not under the support processes of a job counselor. It could be considered that people who attend employment guidance services have a greater commitment to their own insertion besides a greater need to find a job, and this necessity is related to personal self-fulfillment. It is also a reality that unemployed people tend to give a higher assessment of the importance of work, not only because of the values they have acquired, but also because it is the source of other necessary resources to live, for example, the economic income.

As far as relative centrality is concerned, people who attend the employment guidance service give, in order of preference, special relevance to family, work, leisure, community participation, and religion. In the case of people who do not attend the employment guidance services, the order of priorities is family, work, community participation, leisure, and religion. These results coincide with those obtained by García-Montalvo et al. (1999) and Valls and Martínez (2004) in their research carried out with unemployed people. There are also statistically significant differences in both groups, except for the item *religion*. The group of unemployed people who attend the employment guidance services show higher scores in the items work and family. In the items leisure and community participation, the group of participants who do not attend employment guidance obtain higher scores. Other studies carried out with university students highlight the importance of the family, followed by free time and leisure (Aisenson, 2009). This difference in scores could be explained by the general higher level of commitment with regard to those vital issues that are paramount for some people with respect to others. This greater commitment, when related to work and the unemployment situation, will drive them to be more active in their job search, taking advantage of the specific resources existing, among which it is worth highlighting those of employment guidance.

In relation to the dimension *valued work outcomes*, statistically significant differences can be found regarding *advancement and promotion*, *conditions*, and *occupational fulfillment*. It is the group of unemployed people who do not attend the employment guidance services that has higher average scores in the items *good opportunities for advancement and promotion*, *good physical working conditions*, and *occupational fulfillment*. Other authors such as Roe and Ester (1999) believe that the activity of people in work-related contexts such as job search, the role of students in a training course, the performance of a role within an organization, the distribution of time between work and family, etc., depend more on the work values than on the general values; but they also say that the role of general values is important as well.

## Conclusion

In relation to the first objective of this work, to carry out a factorial analysis to reduce data and to find possible homogeneous groups of variables, it can be concluded that, first, the dimension *work goals* allows to reduce and to group data into four categories that encompass homogeneous factors: “*work as a challenge*,” “*working conditions*,” “*development of personal relationships*,” and “*status and prestige*.” Second, it can be said that unemployed people in search of employment prove to have high levels of absolute work centrality; and even so, those who attend employment guidance services have a general trend to a greater work centrality than those who, although being unemployed and wanting to find a job, are not conducting specific guidance processes.

In relation to the relative values that unemployed people who participated in the study assume in their lives, there is no mention of differences in the first priorities established, being *family* the first ranked item, and then *work*. It can be seen, again, that those people who attend employment guidance get a higher score in these two items than those who are not in the process of guidance.

These data indicate the need to establish measures from the public level that allow to reach the unemployed people and groups that do not make use of the services and instruments of employment activation, being these groups the ones in a greater need of them for, among other issues, having a lower level of commitment or being less aware of the possibilities that these actions have toward their job insertion.

The results show the necessity to develop interventions that allow access to those who are unemployed and not attending the active employment policies of the enforcement agencies. Precisely, it is these people who show the lowest level of commitment and distrust toward the actions that promote their labor insertion, and therefore, these actions must be oriented to a greater extent.

## Limitations

The main limitation of this study was to obtain the sample. The collective of unemployed people, in many occasions, is going through a personal process of attrition and distrust toward the employment services and the bureaucratic processes that they entail. The feeling of abandonment of the administrations, and the need to obtain a job, or a decent job, are their principal objectives. Therefore, voluntariness in this type of research, in many cases, involve an overexertion on the part of the unemployed themselves, and of the researchers. Moreover, the centrality and meaning at work, despite being a subject of interest to many researchers, being a topic with many connotations related to values, has led to a proliferation of theoretical studies and dissertations of different constructs, as opposed to the scarcity of empirical work to obtain results that can be compared to other groups.

## Transparency Declaration

The corresponding author on behalf of the other authors guarantees the accuracy, transparency, and honesty of the data and information contained in the study that no relevant information has been omitted and that all discrepancies between authors have been adequately resolved and described.

## Author Contributions

JC-R and JG-S conceived and designed the study, with assistance from YN-A and ML-L. YN-A and JC-R carried out the analyses, supervised by JG-S, with contributions from all other authors. The main versions of the manuscript were written by ML-L and JC-R, with contributions from all other authors. All authors participated in the interpretation of results, approved the final version, and were jointly responsible for adequate revision and discussion of all aspects included in the manuscript.

## Conflict of Interest Statement

The authors declare that the research was conducted in the absence of any commercial or financial relationships that could be construed as a potential conflict of interest.

## References

[B1] Aisenson G. (2009). *Representaciones, Preferencias Y Elecciones Ocupacionales De Los Jóvenes Que Finalizan La Escuela Media.* Doctoral thesis, Universidad de Buenos Aires, Argentina.

[B2] Blanch J. M. (2011). La psicología del trabajo ante la crisis del empleo. *Infocop* 55 7–13.

[B3] Cohen J. (1988). *Statistical Power Analysis For The Behavioral Sciences*, 2nd Edn New Jersey, NJ: Lawrence Erlbaum.

[B4] Dakduk S. Torres C. (2013). Los nuevos significados del trabajo. *Debates IESA* 18 25–28.

[B5] De Pablo-Urban J. M. (1996). Desarrollo de los aspectos personales para la ocupación: una metodología para el cambio con grupos de desempleados. *Interv. Psicosoc.* 15 75–101.

[B6] Dubin R. (1956). Industrial workers’ worlds: A study of the “central life interests” of industrial workers. *Soc. Probl.* 3 131–142. 10.2307/799133

[B7] Dubin R. (1976). *Handbook of Work, Organization and Society.* Chicago: Rand McNally.

[B8] Gallardo J. (2011). Juventud, trabajo, desempleo e identidad: un enfoque psicosocial. *Athenea Digital* 11 165–182.

[B9] García-Montalvo J. Peiró J. M. (1999). *Capital Humano, El Mercado Laboral De Los Jóvenes: Formación, Transición Y Empleo.* Valencia: Bancaja-Ivie.

[B10] García-Montalvo J. Peiró J. M. Soro A. (2003). *Capital Humano, Observatorio De La Inserción Laboral De Los Jóvenes.* Valencia: Bancaja-Ivie

[B11] Gil-Monte P. R. (2003). El síndrome de quemarse por el trabajo (síndrome de burnout) en profesionales de enfermería. *Rev. Eletrôn. InterAção Psy.* 1 19–33.

[B12] Gorz A. (1997). *Metamorfosis Del Trabajo.* Madrid: Sistema.

[B13] Gracia F. Pinazo D. Carrero V. (2001). Implicación, compromiso y centralidad con el trabajo: ¿es o no el mismo concepto? *Revista de Psicología del Trabajo y de las Organizaciones* 17 109–121.

[B14] Hoppock R. Super D. E. (1950). “Vocational and educational satisfaction,” in *Handbook of Applied Psychology*, eds Fryer D. H. Henry E. R. (New York, NY: Rinehart), 126–134.

[B15] Huberty C. J. (2002). A history of effect size indices. *Educ. Psychol. Meas.* 62 227–240. 10.1177/0013164402062002002

[B16] Jiménez I. (2017). Orientación laboral: una revisión bibliográfica de su conceptualización y su aporte a la persona trabajadora y a las organizaciones laborales. *Rev. Electrón. Educare.* 21 1–17. 10.15359/ree.21-2.19 6705090

[B17] Kanungo R. N. (1991). Making meaning of MOW research: a discussion. *Eur. Work Org. Psychol.* 1 161–165. 10.1080/09602009108408520

[B18] McClelland D. C. (1961). *The Achieving Society.* New York: Free Press 10.1037/14359-000

[B19] McClelland D. C. (1976). *Human Motivation.* New York: Cambrige University Press.

[B20] Méda D. (1998). *El trabajo. Un Valor en peligro de Extinción.* Barcelona: Gedisa.

[B21] Montilla S. (2003). Orientación profesional para el empleo: un esquema de trabajo multidimensional. *Revista de Psicología del Trabajo y de las Organizaciones*, 19 25–57. 6705090

[B22] Meaning of Working [MOW] International Research Team (1981). “The Meaning of Working,” in *Management under Differing Value Systems*, eds Dlugos G. Weiermair K. (Berlin: Walter de Gruyter), 565–630.

[B23] Meaning of Working [MOW] International Research Team (1987). *The Meaning of Working.* Londres: Academic Press.

[B24] Offe C. (1992). ¿Es el trabajo una categoría sociológica clave? En C. *Offe et al., La sociedad del trabajo. Problemas estructurales y perspectivas de futuro (pp* 17-51). Madrid: Alianza.

[B25] Panagopoulos C. (2013). Extrinsic rewards, intrinsic motivation and voting. *J. Polit.* 75 266–280. 10.1017/S0022381612001016

[B26] Pérez de las Vacas C. (2015). *Inserción Laboral De Universitarios Desde La Perspectiva Psicosocial.* thesis doctoral, Universidad de Extremadura, España.

[B27] Piqueras R. (2013). *Motivación de Búsqueda de Empleo. En j. Alconada et al., guia de aspectos generales para el desarrollo de las acciones de orientación laboral..* España: Junta de Castilla y León (Consejería de Economía y Empleo) 103–122.

[B28] Pryor R. G. (1981). Interest and values as preferences. *Aust. Psychol.* 16 258–272. 10.1080/00050068108255899

[B29] Reiss S. (2012). Intrinsic and extrinsic motivation. *Teach. Psychol.* 39 152–156. 10.1177/0098628312437704

[B30] Roe R. A. Ester P. (1999). Values and work-Findings and theoretical perspective. *Appl. Psychol. Int. Rev.* 48 1–21. 10.1111/j.1464-0597.1999.tb00046.x

[B31] Ruiz-Quintanilla S. A. Claes R. (2000). “MOW research programs,” in *Databases for The Study of Entrepreneurship*, ed. Katz J. A. (New York, NY: JAI/Elsevier Science Inc.), 335–391. 10.1016/S1074-7540(00)04010-1

[B32] Salanova M. (1992). *Un Estudio Del Significado Del Trabajo En Jóvenes De Primer Empleo.* thesis doctoral, Universidad de Valencia, Valencia.

[B33] Salanova M. Calvo M. Sancerni M. D. González V. (1987). «Significado del Trabajo para los Jóvenes: Adaptación del Cuestionario “Meaning of Working” (MOW, 1981-1987)», Trabajo presentado al II Congreso de Evaluación Psicológica. Madrid.

[B34] Salanova M. Bravo M. J. Hontangas P. Rodríguez I. Peiró J. M. Gracia F. J., (1993). Centralidad del trabajo. *En J. M Peiró, F. Prieto, M. J. Bravo, P. Ripio, I. Rodríguez, P. Hontangas y M. Salanova, Los jóvenes ante el primer empleo, el significado del trabajo y su medida (pp* 55-58). Valencia: Nau Llibres.

[B35] Salanova M. Gracia F. J. Peiró J. M. (1996). Significado del trabajo y valores laborales. *En J. M. Peiró y F. Prieto (Eds), Tratado de Psicología del Trabajo. (pp.*35-63). Madrid. Síntesis.

[B36] Selva J. (1988). *Un Modelo De Socialización Laboral Para El Estudio De La Inserción Laboral Y Las Transiciones En El Sistema Educativo.* thesis doctoral, Universitat de València, Valencia.

[B37] Schwartz S. H. (1992). Universals in the content and structure of values: theory and empirical tests in 20 countries. *Adv. Exp. Soc. Psychol.* 25 1–65. 10.1016/S0065-2601(08)60281-6

[B38] Sverko B. (1989). Origin of individual differences in importance attached to work. A model and a contribution to its evaluation. *J. Vocat. Behav.* 34 28–39. 10.1016/0001-8791(89)90062-6

[B39] Super D. E. (1968). *Work Values Inventory.* Boston: Houghton Mifflin.

[B40] Vanderberg R. Self R. (1993). Assessing newcomers’ changing commitments to the organization during the first 6 months of work. *J. Appl. Psychol.* 78 557–568. 10.1037/0021-9010.78.4.557

[B41] Zytowski D. G. (1970). The concept of work values. *Vocat. Guid. Q.* 18 176–186. 10.1002/j.2164-585X.1970.tb00231.x

[B42] Zigarmi D. Nimon K. Houson D. Witt D. Diehl J. (2009). Beyond engagement: toward a framework and operational definition for employee work passion. *Hum. Res. Dev. Rev.* 8 300–326. 10.1177/1534484309338171

[B43] Valls F. Martínez J. M. (2004). La centralidad y el valor del trabajo en el proceso de inserción laboral de personas desempleadas. *Revista de Psicología del Trabajo y de las Organizaciones* 20 337–354.

